# Metabolomic and Proteomic Profiling of Porcine Intestinal Epithelial Cells Infected with Porcine Epidemic Diarrhea Virus

**DOI:** 10.3390/ijms24065071

**Published:** 2023-03-07

**Authors:** Haifei Wang, Peng Hui, Yoshinobu Uemoto, Yueyun Ding, Zongjun Yin, Wenbin Bao

**Affiliations:** 1 Key Laboratory for Animal Genetics, Breeding, Reproduction and Molecular Design, College of Animal Science and Technology, Yangzhou University, Yangzhou 225009, China; 2 Animal Breeding and Genetics, Graduate School of Agricultural Science, Tohoku University, Sendai 980-8572, Japan; 3 College of Animal Science and Technology, Anhui Agricultural University, Hefei 230036, China; 4 Joint International Research Laboratory of Agriculture and Agri-Product Safety, The Ministry of Education of China, Yangzhou University, Yangzhou 225009, China

**Keywords:** porcine epidemic diarrhea virus, metabolomics, proteomics, virus replication

## Abstract

Porcine epidemic diarrhea virus (PEDV) infection results in severe epidemic diarrhea and the death of suckling pigs. Although new knowledge about the pathogenesis of PEDV has been improved, alterations in metabolic processes and the functional regulators involved in PEDV infection with host cells remain largely unknow. To identify cellular metabolites and proteins related to PEDV pathogenesis, we synergistically investigated the metabolome and proteome profiles of PEDV-infected porcine intestinal epithelial cells by liquid chromatography tandem mass spectrometry and isobaric tags for relative and absolute quantification techniques. We identified 522 differential metabolites in positive and negative ion modes and 295 differentially expressed proteins after PEDV infection. Pathways of cysteine and methionine metabolism, glycine, serine and threonine metabolism, and mineral absorption were significantly enriched by differential metabolites and differentially expressed proteins. The betaine-homocysteine S-methyltransferase (BHMT) was indicated as a potential regulator involved in these metabolic processes. We then knocked down the BHMT gene and observed that down-expression of BHMT obviously decreased copy numbers of PEDV and virus titers (*p* < 0.01). Our findings provide new insights into the metabolic and proteomic profiles in PEDV-infected host cells and contribute to our further understanding of PEDV pathogenesis.

## 1. Introduction

In recent years, porcine epidemic diarrhea (PED) which is caused by porcine epidemic diarrhea virus (PEDV) is still a refractory disease for large scale pig farms. PED was first reported in Europe in the 1970s and then outbreaks were further described worldwide, in many European and Asian countries since the 1990s and in North American in 2013 [1]. PEDV belongs to the genus *Alphacoronavirus* in the family *Coronaviridae* and currently spreads throughout the world as the most prevalent porcine coronaviruses [2]. Contaminated feed, feed ingredient, equipment, aerosols, and other fomites can serve as vehicles for the introduction and transmission of PEDV [1,3]. PEDV enters the intestinal target cells through the fecal–oral and respiratory transmission routes, resulting in dysfunctions of intestinal cells and damages in intestinal barriers [4]. PEDV causes epidemic diarrhea and vomiting which brings substantial losses to the pig industry because of the high morbidity and mortality for suckling pigs. Among the proteins encoding by the PEDV genome, the spike protein plays important roles for virus entry through interactions with receptors expressed in host cells [5]. Furthermore, PEDV is capable of avoiding immune responses of host cells by interfering with the production of interferons and antiviral proteins [6,7]. Given the complicated interactions between host cells and PEDV, it is necessary to investigate their interaction from various layers for a better understanding of PEDV pathogenesis and the efficacious control of PEDV infection.

Metabolomics analysis enables the identification of metabolites that act as drivers of biological processes, and its integration with other omics data such as proteome and transcriptome provides a powerful strategy to depict the regulatory networks of metabolisms [8]. Viral infection alters the metabolic processes of host cells, and the precise metabolic alterations vary with the specific viruses and the types of infected host cells. Glycolysis, lipid synthesis, tricarboxylic acid cycle, and nucleotide biosynthesis are the common metabolic processes hijacked by various viruses [9]. A virus can induce metabolic changes to avoid immune response and promote its replication in host cells. The hepatitis B virus avoids innate immune recognition by activating glycolysis to impede retinoic acid-inducible gene I (RIG-I)-induced interferon production [10]. African swine fever virus infection elevates the host tricarboxylic acid cycle and amino acid metabolism to promote its replication in host cells [11]. All the data indicate the important means for viruses to survive in host cells by rigging the metabolic processes of host cells. PEDV infection results in the physiological dysfunction of intestinal cells, while the metabolic processes associated with PEDV infection and the involved key metabolites remain largely unknown.

In this study, to identify metabolites and proteins related to PEDV infection, we systematically investigated the metabolic and proteomic changes induced by PEDV infection in porcine intestinal epithelial (IPEC-J2) cells. We demonstrated metabolic and proteomic profiling of IPEC-J2 with PEDV infection and revealed the most influenced metabolic processes including cysteine and methionine metabolism, glycine, serine, and threonine metabolism. Furthermore, functional analysis uncovered the roles of betaine-homocysteine S-methyltransferase (BHMT) that engages in the two metabolic processes in regulating PEDV infection.

## 2. Results

### 2.1. Metabolomic Changes Associated with PEDV Infection

**Figure 1:**
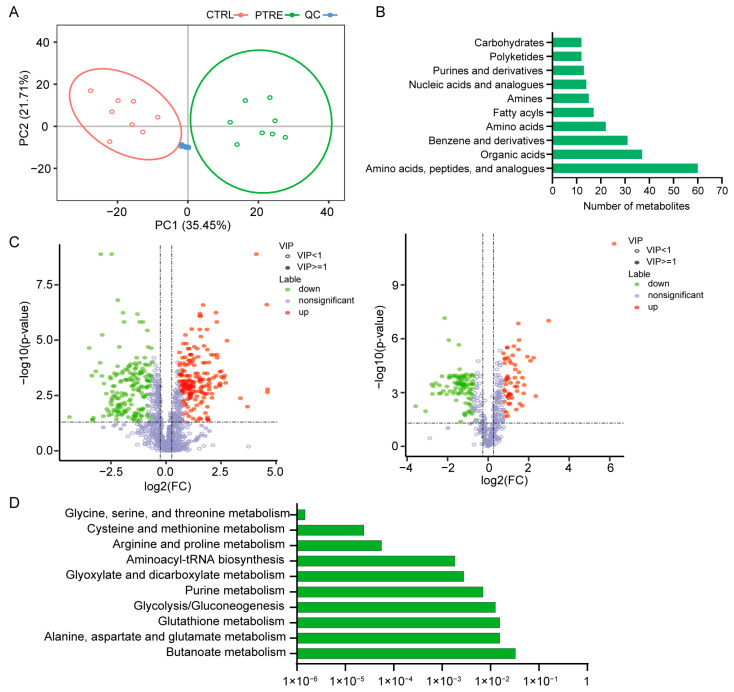
Metabolic changes in cells infected with PEDV. (**A**) Principal component analysis of PEDV-infected and uninfected cell samples. PTRE: PEDV-infected samples; CTRL: uninfected samples; QC: quality control samples. (**B**) Classification of the identified metabolites. (**C**) Volcano plots of differential metabolites in positive (left panel) and negative (right panel) modes between the PEDV-infected and uninfected groups. The red and green dots represent the upregulated and downregulated metabolites. (**D**) Metabolic pathways enriched by the differential metabolites.

To detect the metabolites involved in cellular responses to PEDV infection, metabolomic changes in PEDV-infected cells were analyzed using liquid chromatography tandem mass spectrometry (LC–MS/MS) techniques. Principal component analysis showed obvious separation of the PEDV-infected and uninfected control samples, which indicates significant metabolomic changes post-PEDV infection and high stability of the data analysis processes (Figure 1A). Annotation for metabolites showed that all of the metabolites mainly belonged to 10 categories including amino acids, peptides, and analogues, organic acids, benzene, and derivatives (Figure 1B). The partial least-squares discrimination analysis was then performed to identify the relationships between the metabolite levels in both ion modes and sample types, which indicated obvious separation of the samples from the two groups (Figure S1).

Differential analysis revealed 366 differential metabolites in positive mode, of which 191 metabolites were upregulated and 175 metabolites were downregulated, and 156 differential metabolites in were in negative mode, of which 58 metabolites were upregulated and 98 metabolites were downregulated (Figure 1C; Table S1). To reveal the potential functions of the differential metabolites, we performed pathway enrichment analysis and observed that the metabolic pathways including glycine, serine, and threonine metabolism, cysteine and methionine metabolism, and arginine and proline metabolism were significantly enriched (Figure 1D; Table S2). Hierarchical clustering of differential metabolite was then conducted to display the expression patterns of the metabolites between the PEDV-infected and control groups (Figure S2). The findings indicated that PEDV can induce metabolic changes in host cells and the glycine, serine, and threonine metabolism as well as the cysteine and methionine metabolism were the most affected metabolic processes.

### 2.2. Alterations in Proteome Induced by PEDV Infection

**Figure 2:**
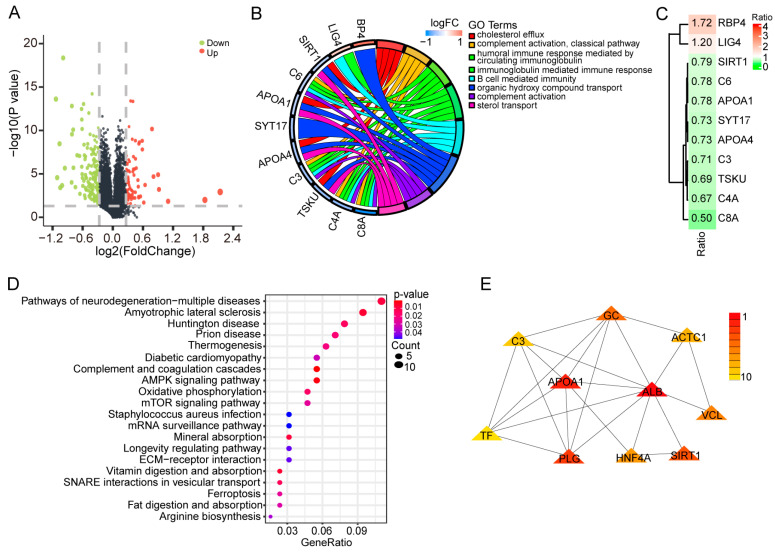
Proteomic analysis of PEDV-infected IPEC-J2 cells. (**A**) Volcano plot of differential expression proteins between the PEDV-infected and uninfected groups. The red and green dots represent upregulated and downregulated proteins after PEDV infection, respectively. (**B**) Functional annotation for differentially expressed proteins. LogFC indicates the log2-transformed fold changes in protein expression between the PEDV-infected and uninfected groups. (**C**) Log2-transformed fold changes in the proteins involved in the functional terms. (**D**) Pathways significantly enriched by the differentially expressed proteins. (**E**) Core proteins identified by interactive analysis based on the STRING database. The coded color bar indicates the ranks of core proteins.

To analyze the alterations in proteins associated with PEDV infection, we performed isobaric tags for relative and absolute quantification (iTRAQ) proteomic analyses using four PEDV-infected samples and four uninfected controls. For all samples, 28,081 unique peptides and 6048 corresponding proteins were identified (Figure S3). Differential analysis revealed 295 differentially expressed proteins between the two groups, of which 95 proteins were upregulated, and 200 proteins were downregulated after PEDV infection (Figure 2A; Table S3). Prediction of subcellular localization showed that a majority of the proteins were located in the nucleus (31.6%) and cytoplasm (21.8%), and some proteins were located in the extra-cell (18.0%) and mitochondrion (11.6%) (Figure S4). Gene ontology analysis showed that the differentially expressed proteins were mainly enriched in the cholesterol efflux, complement activation, and humoral immune response mediated by circulating immunoglobulin (Figure 2B; Table S4). Most of the proteins involved in these biological terms were downregulated (Figure 2C). In addition, pathway analysis showed that the differentially expressed proteins were enriched significantly in the complement and coagulation cascades, AMPK signaling pathway, and vitamin digestion and absorption (Figure 2D; Table S5). The STRING database was then used to construct the interactive networks of differentially expressed proteins (Figure S5), which demonstrates the core proteins including ALB, APOA1, and PLG (Figure 2E). The results demonstrated the proteins whose abundance was significantly affected and indicated their potential functions in regulating PEDV infection.

### 2.3. Integrated Analysis of Metabolome and Proteome Data

**Figure 3:**
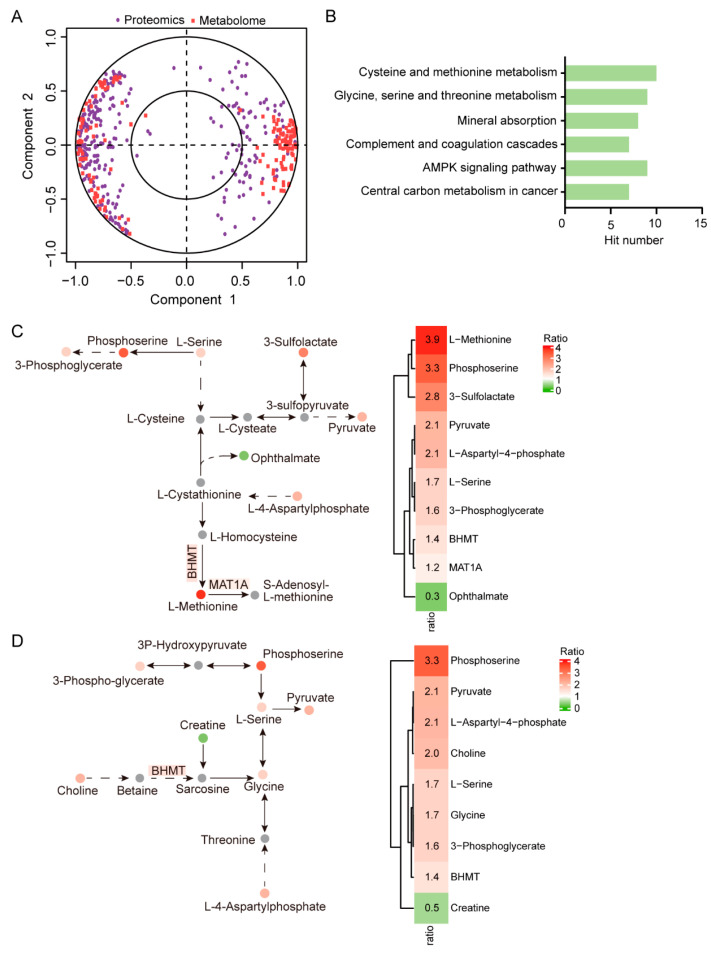
Integrated analysis of metabolome and proteome data. (**A**) Correlation circle plot of differential metabolites and differentially expressed proteins. The included angle (<90 degrees) between a metabolite and protein means positive correlations, and the included angle (>90 degrees) means negative correlations. (**B**) Pathways jointly enriched by differential metabolites and differentially expressed proteins. (**C**) The cysteine and methionine metabolism pathway (**left panel**) and the changes of the involved metabolites (**right panel**). The coded color and corresponding number represent the log2-transformed fold changes in the metabolites. The grey dots indicate non-significant metabolites. (**D**) The glycine, serine, and threonine metabolism pathway (**left panel**) and the changes in involved metabolites (**right panel**). The coded color and corresponding number represent the log2-transformed fold changes in the metabolites. The grey dots indicate non-significant metabolites.

Combined analysis of metabolome and proteome data can provide an additional layer of information for metabolomic and molecular mechanisms involved in physiological or pathological phenotypes. We thus performed integrated analysis of differential metabolites and differentially expressed proteins. The correlations between differential metabolites and differentially expressed proteins are shown in Figure 3A, and the heatmap of correlations is shown in Figure S6 (Table S6). Further enrichment analysis revealed that the differential metabolites and differentially expressed proteins were significantly enriched in pathways including cysteine and methionine metabolism, glycine, serine, and threonine metabolism, and mineral absorption (Figure 3B; Table S7). In addition, most of the metabolites and proteins involved in the top-ranked cysteine and methionine metabolic process (Figure 3C) and the glycine, serine, and threonine metabolic process (Figure 3D) were dramatically upregulated. These observations indicated that changes in the expression levels of these metabolites and proteins may act as a hallmark of host cells infected by PEDV.

### 2.4. Knockdown of BHMT Reduces PEDV Replication in Host Cells

**Figure 4:**
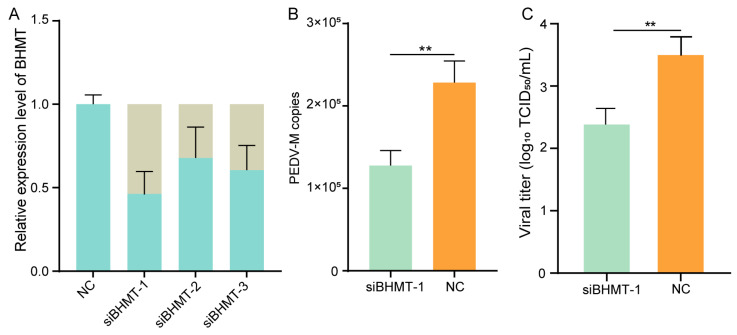
BHMT regulates the infection of PEDV with IPEC-J2 cells. (**A**) Interference efficiency of siRNAs targeting BHMT quantified by qRT-PCR. siBHMT-1, siBHMT-2, and siBHMT-3 indicate three different siRNAs. NC: negative control. The sandy and light blue respectively denote the interference efficiency and expression efficiency of the BHMT gene. (**B**) Genome copy numbers of PEDV in BHMT knockdown and control cells. siBHMT-1: knockdown of BHMT by siRNA siBHMT-1; NC: negative control. (**C**) Viral titer of PEDV in BHMT knockdown and control cells. siBHMT-1: knockdown of BHMT by siRNA siBHMT-1; NC: negative control. Values are presented as mean ± standard derivation. ** *p* < 0.01.

The BHMT protein was involved in the most significantly enriched amino acid metabolic pathways identified by integrative analysis of metabolome and proteome data. BHMT that catalyzes the conversion of betaine and homocysteine to dimethylglycine and methionine is an important enzyme involved in the processes of cysteine and methionine metabolism, and glycine, serine, and threonine metabolism [12]. To explore the potential roles of BHMT in response to PEDV infection, we knocked down the expression of BHMT by RNA interference technique. A significant decrease in BHMT expression was observed by qRT-PCR in BHMT knockdown cells (Figure 4A), of which the siBHMT-1 reached the highest interference efficiency and was used in subsequent experiments. We then infected the BHMT knockdown IPEC-J2 cells with PEDV and found that the genome copy numbers (Figure 4B) and virus titers (Figure 4C) were obviously decreased in BHMT knockdown cells compared with those of control cells. Our findings indicated that BHMT may regulate PEDV replication in host cells through influencing the amino acid metabolic processes.

## 3. Discussion

Integrative analysis of metabolome and proteome data has been proven to be as efficacious strategy to uncover molecular events underlying complex biological processes, and has been utilized in identifying functional proteins and metabolites involved in virus infection [13,14]. Previous studies have reported the proteomic alterations in PEDV-infected IPEC-J2 cells and jejunum derived from PEDV-infected piglets [15,16,17], which also detected the differential expression of APOA1, ALB, and TF proteins that were predicted to have interactions in this study. The data indicated the potential functions of these interacted proteins in response to PEDV infection. Furthermore, we herein synergistically explored the proteomic and metabolic changes in PEDV-infected IPEC-J2 cells, which provides more layers of information and accelerates the identification of functional regulators involved in the responses of host cells to PEDV infection.

Interfering the metabolic processes of host cells is one of the important routines for a virus to promote its replication or escape the host immune response. Our work showed significant alterations in the levels of a subset of metabolites, which indicates the associations of these metabolites with PEDV infection. The aminoacyl-tRNA biosynthesis which is enriched by differential metabolites has been shown as a key player in RNA virus infection [18]. Recent studies have also showed the metabolic processes manipulated by viruses that induce diarrhea. Murine norovirus infection causes significant increases in metabolites in glycolysis, glycolysis and oxidative phosphorylation, and the pentose phosphate pathway that are important factors in determining viral infections [19]. Yang et al. revealed significant changes in metabolomic profiling in the intestine of pigs infected with swine acute diarrhea syndrome coronavirus and found that bile acid is crucial for promoting virus replication [20]. The findings indicated that these viruses can manipulate host cell metabolism to favor its replication and aggravate the severity of diarrhea. Furthermore, the cellular metabolic processes rigged by virus infection may be novel promising therapeutic targets for virus infections.

In this study, we found increased levels of the metabolites involved in cysteine and methionine metabolism, and glycine, serine, and threonine metabolism. Among the identified metabolites, the level of pyruvate was significantly elevated post-PEDV infection. As the end product of glycolysis, pyruvate can be oxidized into acetyl coenzyme A to participate in tricarboxylic acid cycle or metabolized into lactate by lactate dehydrogenase with a lack of oxygen [21]. Increased levels of pyruvate have been detected in many types of virus infection such as the hepatitis B virus and African swine fever virus [10,11], which is utilized for virus replication in host cells. These findings together indicated that PEDV may rig the metabolic processes in which pyruvate acts as the key metabolite to promote its replication in host cells. The complement system is constitutive of innate and acquired immunity and plays diverse roles in antiviral immune responses. It can target and eliminate virus particles, and mark the virus-infected cells for elimination by other routes of the immune response [22]. As the defend system to combat the virus, the complement system is the target attacked by the virus for the establishment of virus infection. We found the downregulation of complement component proteins including C3, C6, C4A, and C8A, which indicates their potential roles in mediating the interactions between PEDV and host cells. C3 is responsible for the amplification of the complement pathway activation and its expression in host cells can be repressed by viruses such as PEDV and hepatitis C virus for their escape from an immune response [7,23]. In addition, transcriptional repression of the two isoforms (C4A and C4B) of C4 was observed in cells infected with hepatitis C virus and flavivirus [24,25]. C6 and C8 are parts of the membrane attack complex that penetrates the targeted cell membranes to cause cell lysis and death [26], the formation of which can also be inhibited by virus infection [27]. These findings indicate that the complement system is likely hijacked by PEDV via interfering the expression of these complement component proteins to promote virus infection in host cells.

BHMT is an enzyme found mainly in the liver and aids cells to convert homocysteine into methionine. Previous studies have demonstrated the relationships BHMT with hepatic virus infections. For example, genetic polymorphism rs585800 of BHMT was associated with the risk of hepatitis B virus infection [28]. A dramatical reduction in the BHMT gene expression was observed in 90% of patients with hepatitis C virus-induced cirrhosis [29]. Our work showed the roles of BHMT in the downregulation of PEDV infection, which expands the knowledge of BHMT involved in the regulation of porcine enteric coronavirus. Furthermore, gene expression analysis indicated NF-κB acting as a repressor for BHMT [30]. We found the upregulation of BHMT expression after PEDV infection. Nonstructural proteins of PEDV can inhibit NF-κB activation for evading host immune responses [31]. These findings indicated the potential mechanism in which PEDV promotes the BHMT expression by inhibiting NF-κB activation, although it still needs further experimental verification.

This study provided the metabolic and proteomic profiling for IPEC-J2 infected with PEDV. To obtain more detailed information on PEDV–host interactions, functional and mechanistic investigations on how PEDV manipulates the metabolic processes, and which metabolite or metabolic pathway directly participants in PEDV replication are required in future studies. In addition, we investigated the roles of BHMT in the regulation of PEDV infection in this study. However, the molecular mechanisms through which BHMT regulates PEDV replication and whether BHMT functions in other virus infections such as the transmissible gastroenteritis virus and porcine Delta coronavirus remain to be further explored.

## 4. Materials and Methods

### 4.1. Cell Sample Preparation

Porcine intestinal epithelial cells (IPEC-J2) were seeded in 6-well plates at a density of 5 × 10^5^ cells/mL and cultured overnight in an incubator at 37 °C with 5% CO_2_. Cells were infected with PEDV (strain CV777) at MOI = 1, and following 2 h of adsorption, the infected cells were washed three times with PBS and incubated for 24 h in fresh medium. The PEDV-infected cells and uninfected cells were washed twice with cold PBS, scraped from the plates, resuspended in 1 mL of PBS, and centrifuged at 1000× *g* for 5 min at 4 °C. Cell pellets were immediately frozen in liquid nitrogen and stored at −80 °C in a refrigerator for further use.

### 4.2. Metabolite Identification by LC–MS/MS

Eight PEDV-infected samples and eight control samples were collected for metabolic analysis. Cell samples were sonicated for 10 min and centrifuged for 5 min at 5000 rpm at 4 °C. The supernatant was then transferred to new tubes for vacuum freeze-drying and resuspended with 10% methanol. The solution was transferred to autosampler vials for LC-MS/MS analysis [32] using the Q Exactive Benchtop Orbitrap Mass Spectrometer (ThermoFisher Scientific, Waltham, MA, USA). Quality control samples were prepared by pooling the same volume of each sample to assess the reproducibility of LC-MS/MS analysis. The raw data were processed with Compound Discoverer 3.1 software for peak extraction, peak alignment, and metabolite identification. Databases including the in-house-developed BMDB, mzCloud (ThermoFisher Scientific, Waltham, MA, USA), and ChemSpider (http://www.chemspider.com/ (accessed on 10 June 2022)) were used in combination for the identification of metabolites. The results of Compound Discoverer 3.1 were further processed for data normalization and signal correction using metaX [33]. Principal component analysis was performed to observe the distribution and separation trend of the two groups of samples. The identified metabolites were classified and annotated by referring to the KEGG (https://www.genome.jp/kegg/pathway.html (accessed on 15 December 2022)) and HMDB (https://hmdb.ca/ (accessed on 20 December 2022)) databases. The metabolites with variable importance in the projection (VIP) ≥ 1, q-value < 0.05, and fold change ≥ 1.2 or ≤0.833 between the two groups were defined as differential metabolites.

### 4.3. Proteomic Analysis by iTRAQ

Four PEDV-infected samples and four uninfected controls were prepared for proteomic analysis. Proteins of the cells were extracted and quantified using the Bradford method for protein quantification. The quality of extracted proteins was checked with 12% SDS-PAGE electrophoresis. Proteins were digested with the trypsin enzyme to obtain peptides. Peptides were then labelled with the iTRAQ labelling reagent and fractionated using the Shimadzu LC-20AB liquid phase system. The peptides separated by liquid phase chromatography were analyzed by LC-MS/MS. The raw data produced by LC-MS/MS analysis were converted into MGF format for data searching. Quality control was performed to determine whether a re-analysis step was required. An automated software IQuant was applied to the quantification of proteins [34]. The proteins with fold change ≥ 1.2 or ≤0.833 and *p*-value < 0.05 were defined as differentially expressed proteins. WoLF PSORT was used to predict the subcellular localization of differentially expressed proteins [35].

### 4.4. Integrated Analysis of Metabolome and Proteome

Correlation analysis between differentially expressed proteins and differential metabolites was performed using the regularized canonical correlation analysis method. Sparse partial least squares discriminant analysis was conducted to integrate the metabolome and proteome data using mixOmics package of R software [36]. Correlation circle plots of differentially expressed proteins and differential metabolites were constructed using the plotVar function in the mixOmics package.

### 4.5. Functional Annotation for Differentially Expressed Proteins and Differential Metabolites

Functional annotation for differentially expressed proteins was performed based on the Gene Ontology (http://geneontology.org/ (accessed on 1 January 2023)) and KEGG resources (https://www.kegg.jp/kegg/pathway.html (accessed on 15 December 2022)). Integrated functional analyses of differentially expressed proteins and differential metabolites was conducted using MetaboAnalyst (https://www.metaboanalyst.ca/ (accessed on 12 July 2022)). The STRING database (https://string-db.org (accessed on 12 August 2022)) was used to construct the protein–protein interaction (PPI) networks of differentially expressed proteins, and the networks were visualized using Cytoscape (https://cytoscape.org (accessed on 7 November 2022)).

### 4.6. Knockdown of BHMT Expression by siRNA

The small interference sequences targeting the coding region of BHMT and the scramble sequences (Table S8) were designed and synthesized by GenePharma Inc. (Suzhou, China). RNA oligonucleotide was diluted with annealing buffer to a final concentration of 50 μM. A mix of 30 μL for each RNA oligonucleotide solution and 15 μL of 5X annealing buffer was created. The mixture was incubated for 1 min at 90 °C, cooled slowly to room temperature, and stored on ice for immediate use. SiRNAs were transfected into IPEC-J2 cells using Lipofectamine 3000 according to the manufacturer’s protocols (Invitrogen, Waltham, MA, USA). After 24 h of transfection, the interference efficiency of each siRNA was determined using quantitative real-time PCR (qRT-PCR).

### 4.7. qRT-PCR

The total RNA of cell samples was isolated using the TRIZOL reagent (Thermo Scientific, MA, USA). cDNA was synthesized from total RNA using the HiScript II 1st Strand cDNA Synthesis Kit following the manufacturer’s protocols (Vazyme Biotech Co., Ltd., Nanjing, China). Random hexamers were used in cDNA synthesis. The qRT-PCR reaction system consisted of 1 μL cDNA, 10 μL SYBR Green Mixture, 1 μL of each forward and reverse primer, 0.4 μL 50× ROX Reference Dye II, and 6.6 μL deionized water. The thermal conditions were as follows: 95 °C for 15 s, 40 cycles of 95 °C for 5 s, 60 °C for 30 s. The GAPDH gene was used as internal reference genes. The primers of BHMT were designed by using Primer Premier 5.0, and the primers of PEDV M and GAPDH genes were used in previous studies [37]. The primer sequences for the genes of BHMT, PEDV M, and GAPDH are listed in Table S9. Each sample was performed in triplicate, and the 2^−ΔΔCt^ method was used to quantify gene expression levels.

### 4.8. Titration of PEDV

The culture supernatants of the PEDV-infected BHMT knockdown cells were collected after 24 h of infection and serially diluted at a 10-fold (10^−1^~10^−7^) dilution. Vero cells were seeded into 96-well plates and added with 100 μL of the serial dilutions of PEDV. PEDV-infected cells were cultured for 6 days and the cytopathic effect was observed every day. Viral titers were quantified by a 50% tissue culture infectious dose (TCID_50_) using the Reed–Muench method.

### 4.9. Statistical Analysis

The Student’s *t*-test was used to compare the significant differences between PEDV-infected and control groups. All data are presented as the mean ± standard derivation (SD). Statistical significance is shown as follows: * *p* < 0.05, ** *p* < 0.01.

## 5. Conclusions

In conclusion, this study revealed the alterations in metabolome and proteome profiles of IPEC-J2 cells infected with PEDV. The highly interacted metabolic processes of cysteine and methionine metabolism and glycine, serine, and threonine metabolism were identified. Furthermore, the enzyme BHMT involved in the two metabolic processes was found to regulate PEDV replication in host cells. Our study provided new insight into the interactions between PEDV and host cell metabolism and contributed to our further understanding of PEDV pathogenesis.

## Data Availability

The presented data are available upon request.

## Supplementary Materials

The following supporting information can be downloaded at: https://www.mdpi.com/article/10.3390/ijms24065071/s1.
