# Supplementary material for: Metabolomic and Proteomic Profiling of Porcine Intestinal Epithelial Cells Infected with Porcine Epidemic Diarrhea Virus
Source: Int J Mol Sci. 2023 Mar 7;24(6):5071. doi: 10.3390/ijms24065071 (PMC10049511; doi:10.3390/ijms24065071)
Supplement: Supplementary file 1 [file ijms-24-05071-s001.zip › Supplementary figures.pdf]

Supplementary figures

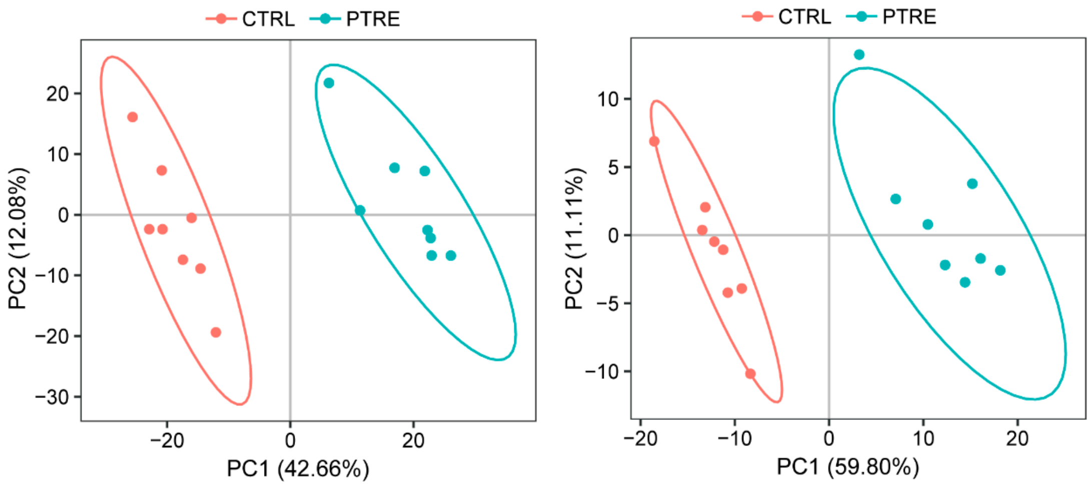

Figure S1. Cluster analysis according to the metabolites in positive (left panel) and negative (right panel) ion modes using the partial least square discrimination method. The red and cyan dots represent control and PEDV-infected samples, respectively.

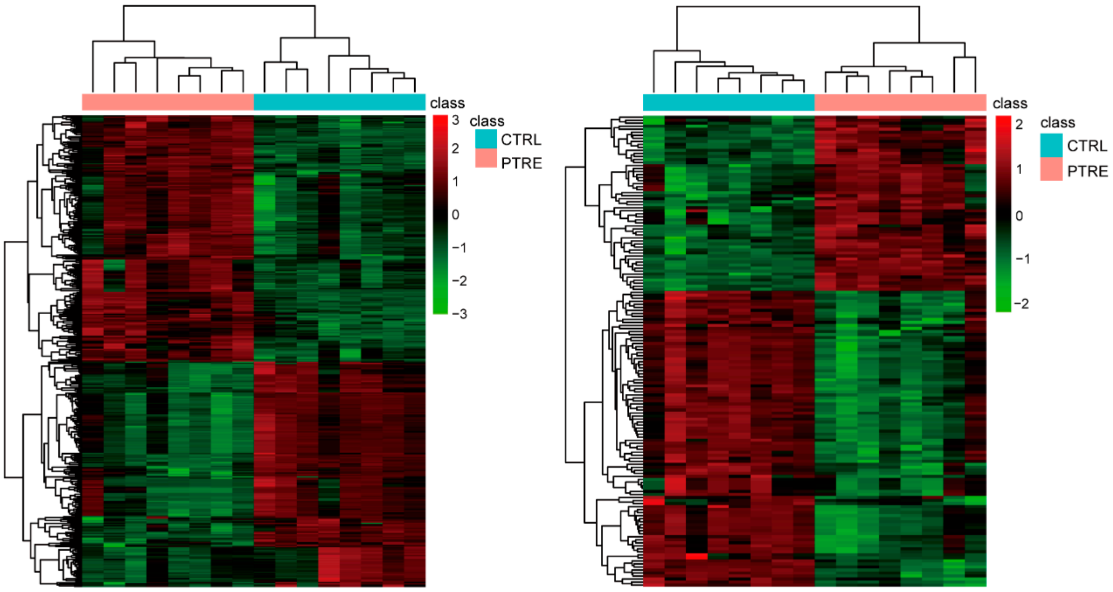

Figure S2. Hierarchical clustering analysis based on the differential metabolites in positive (left panel) and negative (right panel) ion modes between the PEDV-infected and control groups.

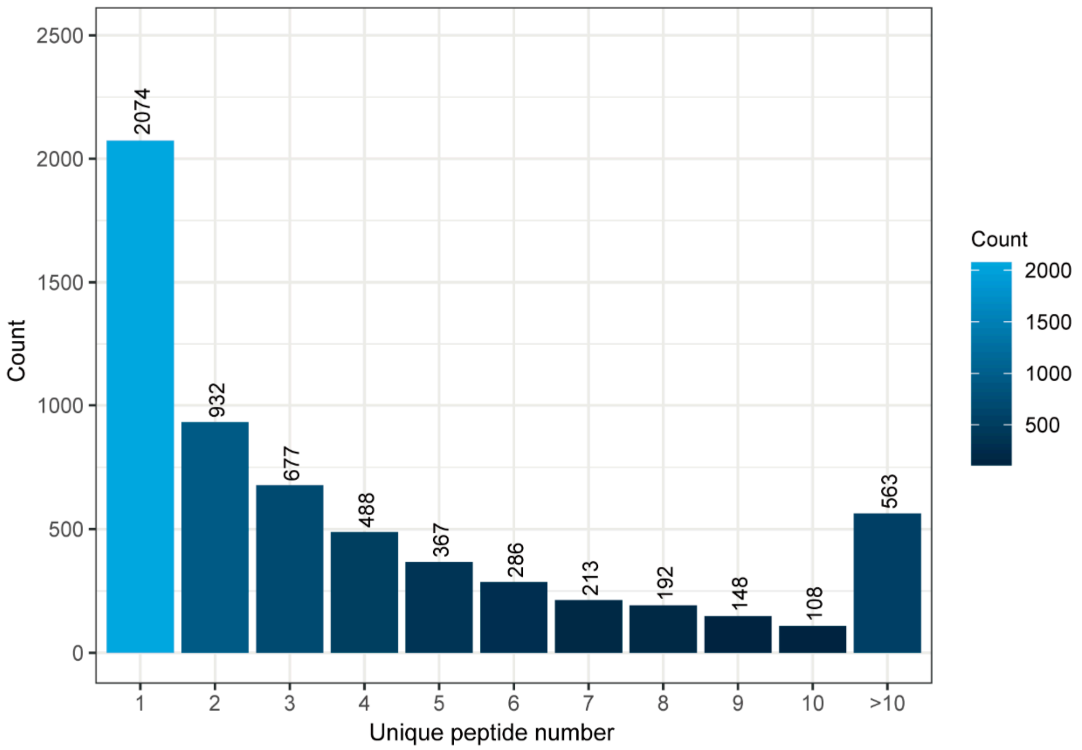

Figure S3. Distribution of the unique peptide number. The X axis indicates unique peptide number, and Y axis indicates protein count corresponding to the unique peptide number. The more unique peptides indicate the higher credibility of protein identification.

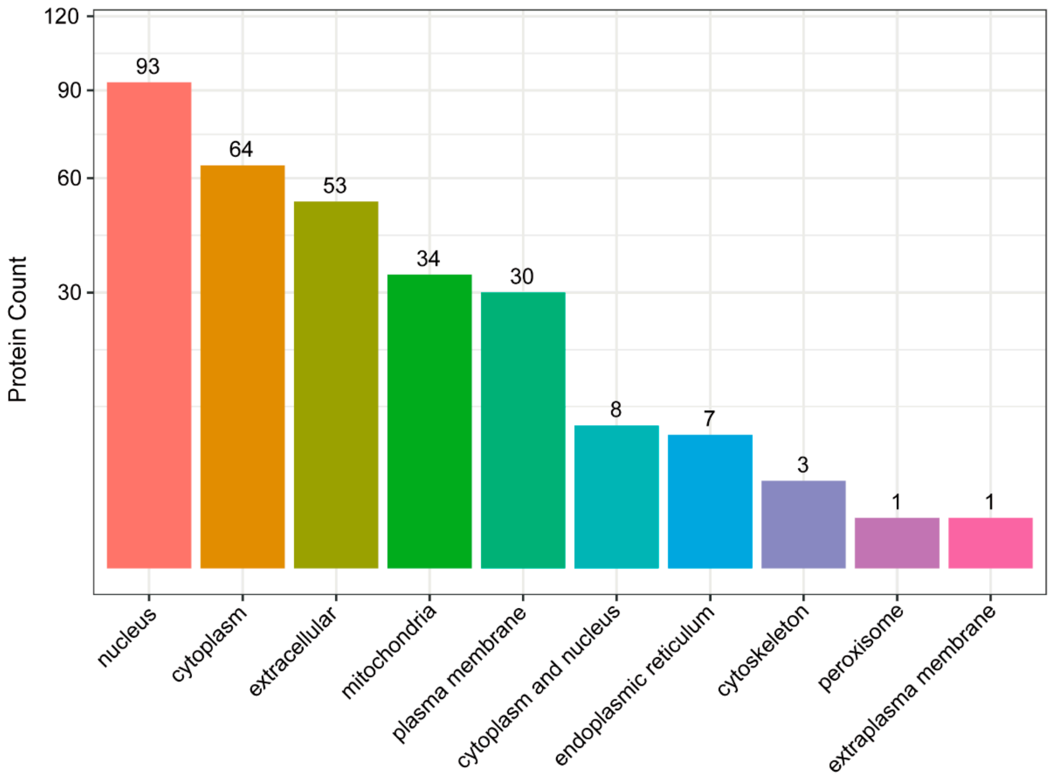

Figure S4. Prediction of subcellular localization of the identified proteins.

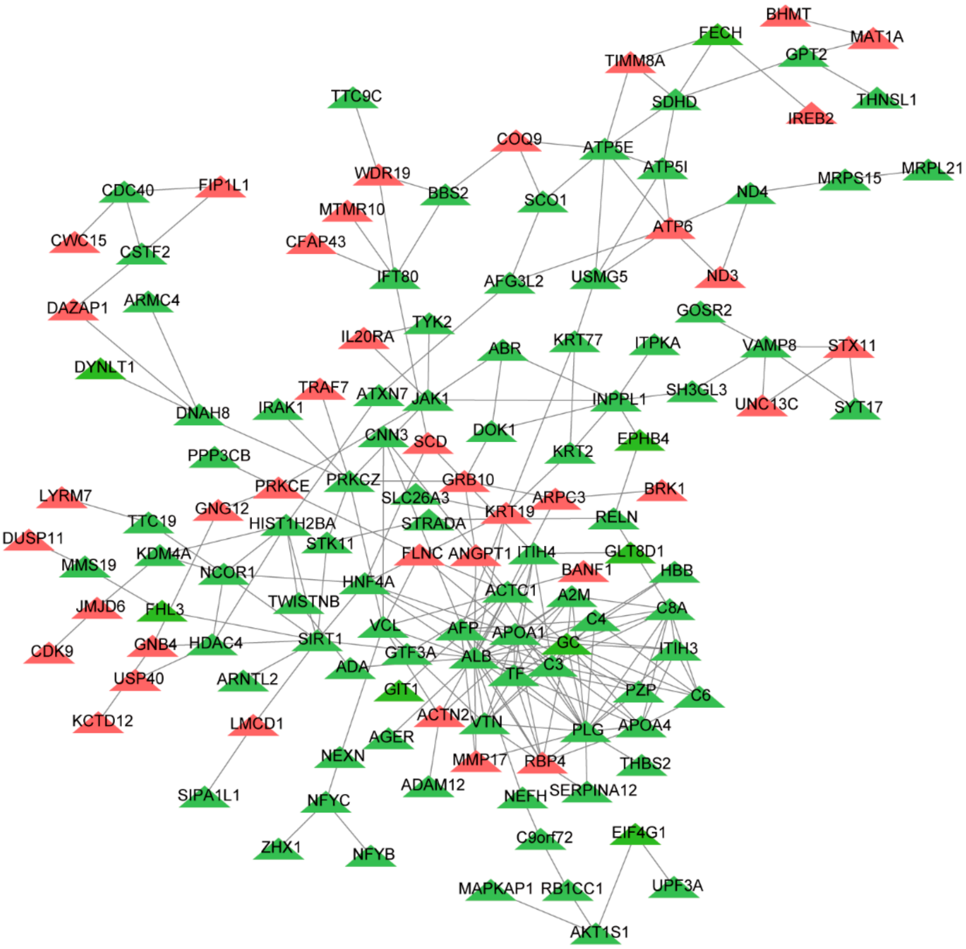

Figure S5. Interactive networks of differentially expressed proteins predicted by the STRING database. The red and green triangle separately represent the upregulated and downregulated protein after PEDV infection.

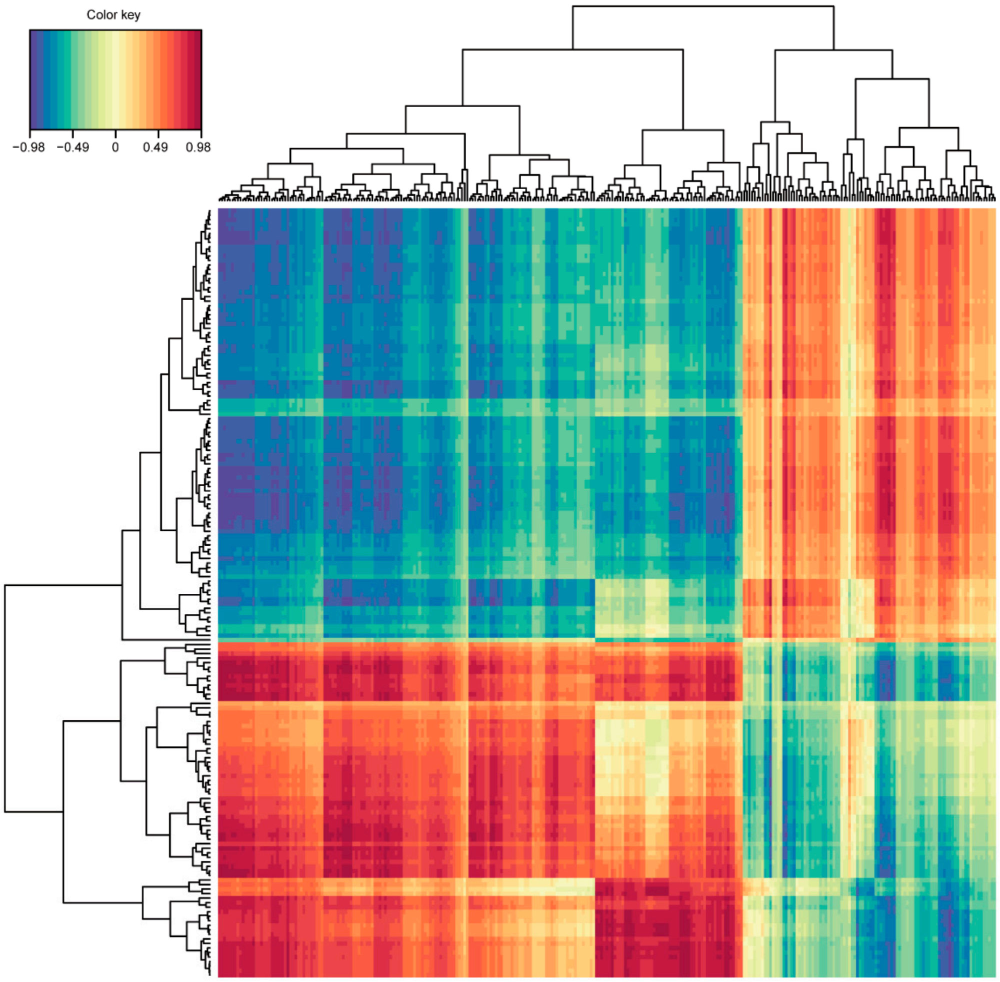

Figure S6. Heatmap of correlations between differential metabolites and differentially expressed proteins between the PEDV-infected and control groups. Each row indicates a differential metabolite and each column indicates a differentially expressed proteins. The color key indicates different correlation coefficients.
